# Longitudinal wearable sensor data enhance precision of Long COVID detection

**DOI:** 10.1371/journal.pdig.0001093

**Published:** 2025-11-20

**Authors:** Chibuike K. Uwakwe, Ekanath Srihari Rangan, Satyajit Kumar, Georg Gutjahr, Xuhui Miao, Andrew W. Brooks, Peter Maguire, Tejaswini Mishra, Lettie McGuire, Michael P. Snyder

**Affiliations:** 1 Department of Genetics, Stanford University School of Medicine, Stanford University, Stanford, California, United States of America; 2 Medical Scientist Training Program, Stanford University School of Medicine, Stanford University, Stanford, California, United States of America; 3 Department of Medicine, Stanford University School of Medicine, Stanford, California, United States of America; 4 Department of Health Science Research, School of Medicine, Amrita Vishwa Vidyapeetham University, Kochi, Kerala, India; 5 Department of Computer Science, School of Engineering, Stanford University, Stanford, California, United States of America; Birjand University of Medical Sciences, IRAN, ISLAMIC REPUBLIC OF

## Abstract

Despite the millions of individuals struggling with persistent symptoms, Long COVID has remained difficult to diagnose due to limited objective biomarkers, often leading to underdiagnosis or even misdiagnosis. To bridge this gap, we investigated the potential of utilizing wearable sensor data to aid in the diagnosis of Long COVID. We analyzed longitudinal heart rate (HR) data from 126 individuals with acute SARS-CoV-2 infections to develop machine learning models capable of predicting Long COVID status using derived HR features, symptom features, or a combination of both feature sets. The HR features were derived across six analytical categories, including time-domain, Poincaré nonlinear, raw signal, Kullback-Leibler (KL) divergence, variational mode decomposition (VMD), and the Shannon energy envelope (SEE), enabling the capture of heart rate dynamics over various temporal scales and the quantification of day-to-day shifts in HR distributions. The symptom features used in the final models included chest pain, vomiting, excessive sweating, memory loss, brain fog, heart palpitations, and loss of smell. The combined HR- and symptom-feature model demonstrated robust predictive performance, achieving an area under the Receiver Operating Characteristic curve (ROC-AUC) of 95.1% and an area under the Precision-Recall curve (PR-AUC) of 85.9%. These values represent a significant improvement of approximately 5% in both the ROC-AUC and PR-AUC over the symptoms-only model. At the population level, this improvement in discrimination could lead to clinically meaningful reductions in misclassification and improved patient outcomes, achieved through a non-invasive diagnostic tool. These findings suggest that wearable HR data could be used to derive an objective biomarker for Long COVID, thereby enhancing diagnostic precision.

## Author summary

Despite the millions of individuals struggling with persistent symptoms, Long COVID has remained difficult to diagnose due to limited objective biomarkers, often leading to underdiagnosis or even misdiagnosis. Wearable devices have recently emerged as powerful tools for real-time health monitoring of body functions, including heart rate (HR). We investigated the utility of wearable HR data collected continuously over an extended period for identifying Long COVID patients. We collected both smartwatch data as well as daily/periodic symptom surveys from participants who had a prior SARS-CoV-2 infection. We used these data from the acute infection period to build machine learning models that identify those who will experience chronic symptoms of Long COVID. When wearable HR data and symptom data are combined into a single model, the predictive performance improves significantly over models using HR data or symptom data alone. We propose a workflow for how a clinician might use our machine learning model to aid clinical diagnosis. Overall, our findings suggest that wearable HR data could be used to derive an objective biomarker for Long COVID, thereby enhancing diagnostic precision.

## Introduction

After an acute SARS-CoV-2 infection, it is estimated that up to 12% of vaccinated individuals and up to 35% of non-hospitalized, unvaccinated individuals develop Long COVID, with symptoms persisting or recurring for at least 3 months [1–3]. Long COVID is a chronic condition arising after SARS-CoV-2 infection characterized by persistent, episodic, or progressive symptoms affecting various organ systems [4,5]. This condition encompasses a wide range of symptoms, including fatigue, dyspnea, and neurological impairments — all of which vary in severity and duration [6–8].

The heterogeneous clinical presentation of Long COVID complicates diagnosis, often leaving patients undiagnosed or misdiagnosed (e.g., as a psychiatric illness), given the absence of an agreed-upon biomarker such as a laboratory test or imaging study [9]. Existing diagnostic frameworks instead rely solely on clinical histories based on self-reported symptoms, which: (i) are inherently subjective, leading to variability in physician responses from patient to patient, and (ii) often resemble other autonomic or psychological disorders, increasing the risk of misdiagnosis [10]. The identification of quantitative biomarkers of Long COVID would greatly facilitate both diagnosis as well as the evaluation of treatment responses.

Wearable devices have recently emerged as powerful tools for real-time health monitoring [11,12]. By continuously measuring a variety of objective physiological metrics — such as heart rate (HR), heart rate variability (HRV), and physical activity levels — these devices offer unique opportunities for early detection and personalized management of chronic conditions, such as cardiometabolic disease, arrhythmias, Chronic Obstructive Pulmonary Disease (COPD), mental health disorders, and even genetic disorders like Friedreich’s ataxia [13–17]. Similar methods have also been used to predict the onset of disease, particularly infections like COVID-19, demonstrating the power of such devices [18,19]. While previous studies have characterized physiological patterns associated with Long COVID, the predictive potential of comprehensive features from wearable data remains largely underexplored [20–24]. Integrating these metrics derived from the acute SARS-CoV-2 infection period with machine learning models could provide actionable insights into the progression to Long COVID, bridging the gap between wearable technology and clinical decision-making and ultimately informing management strategies [25].

The aim of this study was to evaluate the utility of wearable data in association with symptoms during the acute and post-acute infection phases for increasing the precision of Long COVID diagnosis. By leveraging longitudinal sensor data, feature extraction, and machine learning techniques, we demonstrate that commercial wearable devices can significantly assist in detecting Long COVID, paving the way for the development of tools to aid physicians in identifying and managing Long COVID patients effectively.

## Results

**Fig 1:**
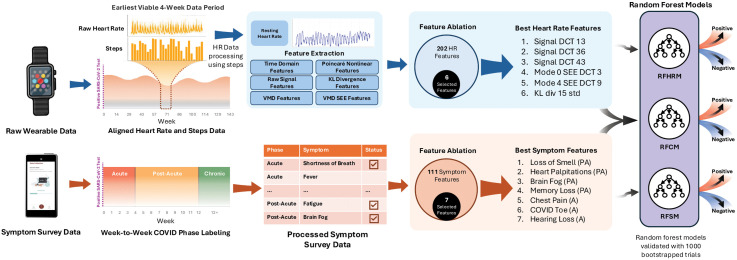
Model architecture and data flow. A schematic illustrating the processing pipeline of wearable and symptom data from individuals post-SARS-CoV-2 infection and the architecture of three models: a Random Forest Heart Rate Model (RFHRM), a Random Forest Symptoms Model (RFSM), and a Random Forest Combined Model (RFCM).

Of the 3,221 total participants in the Stanford COVID wearable study with complete data, 554 had a confirmed acute SARS-CoV-2 infection (Methods) [11,19]. These participants subsequently completed a comprehensive cross-sectional COVID-19 survey that included detailed questions about their symptoms and progression (S1 File; S1 Fig). Importantly, individuals also participated in longitudinal daily surveys, logging their symptom profiles during the acute phase and throughout the course of their illness. The two sources of self-reported symptom data were corroborated with each other to maximize reliability. A subset of 126 participants — 31 with Long COVID and 95 without Long COVID — were used for the machine learning analysis after excluding participants without any 4-week period of continuous HR data (Fig 1).

Raw wearable data, including HR and step counts, were analyzed over a period of 124 weeks post-SARS-CoV-2 infection. Data from each participant’s earliest viable four-week period following their initial COVID-19 diagnosis were preprocessed to mitigate noise from physical activity by excluding HR samples occurring within 30 minutes of any recorded steps to obtain the resting heart rate. Hereafter, resting heart rate is referred to as heart rate (HR). The most commonly used four-week period was weeks 0–4 (S2 Fig; S2 File). From HR we extracted features across six analytical categories: time-domain analysis, raw signal analysis, variational mode decomposition (VMD), Poincaré nonlinear analysis, Kullback-Leibler (KL) divergence, and Shannon energy envelope (SEE). This approach yielded a total of 202 HR features (S3 File). Concurrently, symptom survey data were uploaded to the cloud and processed, with each symptom categorized as acute (within the first 4 weeks after COVID-19 diagnosis), post-acute (between 4–12 weeks after COVID-19 diagnosis), or chronic (12 weeks or more after COVID-19 diagnosis) (S1 File). These symptom phases were defined according to the COVID-19 guideline developed by the National Institute for Health and Care Excellence [26]. This process resulted in the identification of 111 symptom features. After running a feature ablation to glean the most important features, both HR and symptom features were used to train three distinct random forest models — a Random Forest Heart Rate Model (RFHRM), a Random Forest Symptoms Model (RFSM), and a Random Forest Combined Model (RFCM) — and validated with 1,000 bootstrapped trials to detect Long COVID.

**Fig 2:**
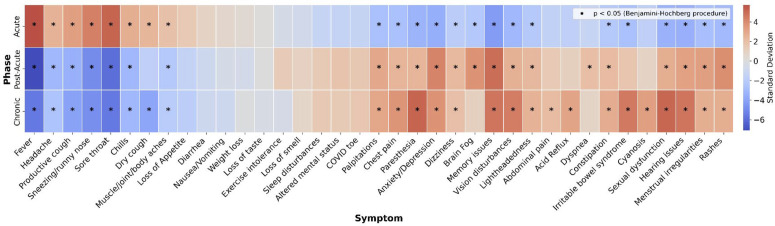
Statistical divergence of symptom prevalence across COVID-19 phases. A heatmap displaying symptoms that were disproportionately reported in each COVID-19 phase (Acute = first 4 weeks, Post-Acute = 4 to 12 weeks, Chronic = after 12 weeks). Markers indicate statistically significant phase-symptom deviations from zero using the Benjamini-Hochberg procedure with a false discovery rate at q = 0.05.

Before constructing the detection models, it was important to characterize the progression of COVID-19 symptoms from the acute phase to the chronic phase. Using the participants’ responses from the cross-sectional COVID-19 survey, we examined symptom profiles across different phases of the disease and identified symptoms that were disproportionately reported in the acute, post-acute, and chronic phases (Fig 2). The symptoms that were disproportionately reported in the acute phase (e.g., fever, headache, etc.) were generally distinct from those that were disproportionately reported during the post-acute and chronic phases, as expected. By comparison, the symptoms that were disproportionately reported in the post-acute and chronic phases corresponded closely (e.g., memory issues, lightheadedness, etc.).

Table 1 describes the cohort characteristics for the 126 participants included in the machine learning analysis. In this cohort, we found that younger participants were significantly more likely to have Long COVID (*P* = 0.00066). Additionally, our data suggest a potential association between female sex and Long COVID (*P* = 0.0070), although this finding did not reach statistical significance after applying a Benjamini-Hochberg correction. For the 31 participants with Long COVID, the median duration of symptoms reported was 90 weeks (IQR: 53–152 weeks; range: 13–260 weeks), highlighting the long-term burden of symptoms captured in this cohort (S3 Fig).

**Table 1:** Characteristics of participants by those with and without Long COVID, n = 126.

Long COVID	No	Yes	*p*-value
n (%)	95 (75.3%)	31 (24.7%)	
Age, mean (SD)	54.44 (11.74)	45.65 (13.28)	0.00066*
Sex			0.0070
Male (%)	53 (55.79%)	8 (25.81%)	
Female (%)	42 (44.21%)	23 (74.19%)	
BMI, mean (SD)	27.82 (6.18)	25.54 (5.95)	0.082
Race/Ethnicity			
European, Caucasian, or White (%)	84.0 (88.42%)	23.0 (74.19%)	0.10
Black or African-American (%)	1.0 (1.05%)	3.0 (9.68%)	0.074
Native American or Alaska Native (%)	0.0 (0.00%)	0.0 (0.00%)	
Asian (%)	5.0 (5.26%)	1.0 (3.23%)	1.0
Native Hawaiian or Pacific Islander (%)	0.0 (0.00%)	0.0 (0.00%)	
Hispanic (%)	2.0 (2.11%)	3.0 (9.68%)	0.18
Other (%)	1.0 (1.05%)	1.0 (3.23%)	0.99
Unreported (%)	2.0 (2.11%)	0.0 (0.00%)	1.0
Condition			
Diabetes (%)	1.0 (3.45%)	0.0 (0.00%)	1.0
Allergy or Autoimmune Disease (%)	6.0 (20.69%)	4.0 (30.77%)	0.75
Cardiovascular Disease (%)	1.0 (3.45%)	0.0 (0.00%)	1.0
Hypertension (%)	7.0 (24.14%)	1.0 (7.69%)	0.41
Hypercholesterolemia (%)	9.0 (31.03%)	4.0 (30.77%)	1.0
Respiratory or Lung Disease (%)	1.0 (3.45%)	3.0 (23.08%)	0.15
Gastrointestinal Disease (%)	3.0 (10.34%)	1.0 (7.69%)	1.0
Hematologic Disease (%)	2.0 (6.90%)	0.0 (0.00%)	0.85
Neurologic Disorder (%)	0.0 (0.00%)	1.0 (7.69%)	0.68
Psychiatric Illness (%)	2.0 (6.90%)	2.0 (15.38%)	0.77
Endocrine Disease (%)	3.0 (10.34%)	2.0 (15.38%)	1.0
Musculoskeletal Disorder (%)	1.0 (3.45%)	0.0 (0.00%)	1.0
Ear, Nose, Throat Disease (%)	0.0 (0.00%)	1.0 (7.69%)	0.68
Genitourinary Disease (%)	1.0 (3.45%)	0.0 (0.00%)	1.0
Dermatologic Condition (%)	1.0 (3.45%)	2.0 (15.38%)	0.46
Lyme Disease (%)	0.0 (0.00%)	1.0 (7.69%)	0.68
Other (%)	0.0 (0.00%)	3.0 (23.08%)	0.042
None (%)	10.0 (34.48%)	3.0 (23.08%)	0.71
Vaccination Status			
Completed 2 doses of vaccine prior to SARS-CoV-2 infection	81.0 (85.26%)	21.0 (67.74%)	0.058

A table displaying the characteristics of study participants used for the machine learning analysis, including age, gender, BMI, race/ethnicity, condition, and vaccination status.

*Statistical significance (q < 0.05) after Benjamini-Hochberg procedure

**Fig 3:**
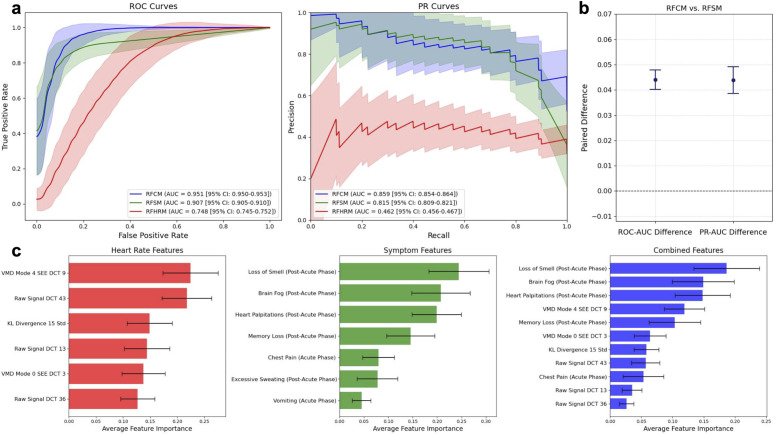
Performance evaluation of random forest models with key features. **(a)** Average ROC curves and PR curves for the Random Forest Combined Model (RFCM), Random Forest Symptoms Model (RFSM), and Random Forest Heart Rate Model (RFHRM). Error bands indicate the standard deviation of the curves. The legends include the mean AUC for each curve and their corresponding 95% confidence intervals. **(b)** The paired difference between the mean areas under the ROC curve (ROC-AUC) and mean areas under the PR curve (PR-AUC) for the RFCM and RFSM. Error bars indicate the 95% confidence intervals for each paired difference. **(c)** Bar graphs showing the average importances for the features in each classifier. Error bars indicate the standard deviation of the average importances. HR features include Raw Signal Discrete Cosine Transform (DCT) 43, Variational Mode Decomposition (VMD) Mode 4 Shannon Energy Envelope (SEE) DCT 9, VMD Mode 0 SEE DCT 3, Kullback-Leibler (KL) Divergence 15 Standard Deviation (Std), Raw Signal DCT 13, and Raw Signal DCT 36.

We constructed three models: 1) RFHRM using only selected HR features, 2) RFSM using only selected symptom features, and 3) RFCM using both selected HR and symptom features. For the RFCM, the relative importances of the symptom features generally outweighed the HR features, though the model made approximately similar use of all the HR features (Fig 3c). Feature importance reflects the average impurity reduction a feature achieves across trees in the random forest model. A high importance score means the feature is effective at splitting nodes into pure groups, improving prediction accuracy.

To evaluate the models, we conducted 1,000 bootstrapped trials in which we randomized the dataset with replacement, using a train-test split of 70% and 30%, respectively. For each of the 1,000 trials, we initialized new classifiers and recorded the Receiver Operating Characteristic (ROC) curve and Precision-Recall (PR) curve (S4 File). Fig 3a shows the aggregate of the ROC curves and the Precision-Recall curves for all trials. The RFHRM achieved a mean area under the ROC curve (ROC-AUC) of 0.748 (95% CI: 0.745–0.752) and a mean area under the PR curve (PR-AUC) of 0.462 (95% CI: 0.456–0.467) (Fig 3a). For the RFSM, the mean ROC-AUC was 0.907 (95% CI: 0.905–0.910), while the mean PR-AUC was 0.815 (95% CI: 0.809–0.821). The RFCM demonstrated a mean ROC-AUC of 0.951 (95% CI: 0.950–0.953) and a mean PR-AUC of 0.859 (95% CI: 0.854–0.864).

Given the proximity of the ROC-AUC and PR-AUC values for the RFCM and RFSM, we conducted a comparative analysis of the two models. Across 1,000 bootstrapped iterations, we computed the paired differences in ROC-AUC and PR-AUC for each model. The mean paired difference in ROC-AUC was 0.044 (95% CI: 0.040-0.048), and the mean paired difference in PR-AUC was 0.044 (95% CI: 0.039-0.049) (Fig 3b).

To evaluate the risk of model overfitting, we compared the performance metrics of each model using the training and test datasets (S4 Fig). The RFCM and RFSM demonstrated consistent performance between training and testing, suggesting robust generalizability. The RFHRM demonstrated substantially higher training performance relative to testing performance, indicating that HR features alone were prone to overfitting in our dataset.

To explore the potential advantages of the RFCM compared to the RFSM, we conducted a case study in which a representative example of each classifier was trained on the same training set (n = 88) and evaluated on a shared test set (n = 38). In this case study, the sensitivity for Long COVID improved from 70% (RFSM) to 100% (RFCM) with a modest drop in specificity from 96% (RFSM) to 93% (RFCM) when HR features were included (S5 Fig). Interpretability is critical for deploying machine learning models in healthcare, especially when they rely on complex features derived from wearable data [27–29]. To explore the interpretability of the RFCM, we performed a SHapley Additive exPlanations (SHAP) analysis on the participants in the case study that were misclassified as non-Long COVID by the RFSM but correctly classified as Long COVID by the RFCM (S6 Fig). This analysis highlighted how HR features contributed to improved classification in the case study, with low values for Variational Mode Decomposition (VMD) Mode 0 Shannon Energy Envelope (SEE) DCT 3, Raw Signal DCT 43, and VMD Mode 4 SEE DCT 9 emerging as strong predictors of Long COVID.

## Discussion

In this study, we investigated the utility of longitudinal wearable data in distinguishing individuals who develop Long COVID from those who do not. We constructed three Random Forest classifiers to evaluate classification accuracy using HR data alone, symptom data alone, and a combination of HR and symptom data.

The most important HR feature selected for the combined-features classifier was Variational Mode Decomposition (VMD) Mode 4 Shannon Energy Envelope (SEE) Discrete Cosine Transform (DCT) 9 (Fig 3c). This feature was derived by taking the highest band of frequencies (VMD Mode 4) and examining the peaks of the signal using its SEE. Analyzing the 9th coefficient of the DCT implies that the periodicity of the signal peaks every 3 days is most informative. This suggests that the periodic behavior of the Mode 4 energy envelope derived from HR was particularly influential in how the RFCM distinguished between Long COVID and non-Long COVID cases.

The most important symptom features selected for the combined-features classifier were: 1) post-acute loss of smell, 2) post-acute brain fog, 3) post-acute heart palpitations, 4) post-acute memory issues, and 5) acute chest pain. All the post-acute symptoms prioritized by the model, except for loss of smell, were significantly overrepresented during this phase (*P* < 0.05) (Fig 1). Conversely, chest pain was significantly underrepresented in the acute phase. These findings suggest that symptom patterns in the post-acute phase, as well as deviations from expected acute symptom profiles, may be critical for identifying individuals at risk for developing Long COVID.

Incorporating both HR features with symptom-derived features into the RFCM yielded a mean ROC-AUC of 95.1%. Due to the imbalanced nature of the dataset used to train the classifiers — 75.3% non-Long COVID and 24.7% Long COVID — the PR-AUC provides a more informative measure of performance compared to the ROC-AUC. The RFCM achieved a mean PR-AUC of 85.9%, indicating a high level of precision in identifying true positive cases of Long COVID relative to the overall number of positive predictions. These metrics reflect a significant improvement of approximately 5% for the ROC-AUC and PR-AUC of the RFCM compared to the RFSM, which achieved a mean ROC-AUC of 90.7% and a mean PR-AUC of 81.5%. This significant improvement in model performance when HR features are used in combination with symptom features suggests that HR data have utility in diagnosing Long COVID, particularly when an individual’s symptom profile is nonspecific. Our overfitting analysis showed that the RFHRM was particularly prone to overfitting compared to the RFCM, which supports the value of multimodal integration of symptoms and HR to develop generalizable models. With more than 750 million cases of COVID-19 reported worldwide, this 5% improvement could substantially reduce false negatives at the population level [30]. This is particularly relevant in the context of Long COVID, where underdiagnosis can delay supportive care and management.

There are several important limitations of this study, which could inform future research in this area. First, the relatively small sample size (n = 126) and the class imbalance in our dataset may limit the generalizability of our findings to broader populations. To address this, we employed model validation techniques and evaluated model performance using both the PR-AUC and the standard ROC-AUC. Our findings may have been influenced by unmeasured confounding variables, including socioeconomic status, medication use (e.g., beta blockers), sleep patterns, and the infecting SARS-CoV-2 variant. The symptom data and Long COVID statuses were self-reported in a survey and therefore are dependent on the accuracy of the participants’ memory and may be subject to recall and reporting bias. While the difference in vaccination rates between the Long COVID and non-Long COVID groups was not statistically significant (*P* = 0.058), the omission of vaccination status from the models’ inputs may have introduced residual confounding given that vaccination status can modulate physiological data patterns. Incorporating vaccination status along with other confounding variables and mitigating recall bias in symptom reporting would enhance the methodological rigor of future analyses. There was a considerable ethnic imbalance among the participants whose data were used to train the models, with approximately 85% of participants identifying as European, Caucasian, or White (Table 1). This imbalance may reduce the performance and accuracy of the model when it is applied to individuals from minority ethnic groups. Expanding this approach to larger, more balanced datasets would strengthen our findings and further validate the utility of wearable data.

This study also presents ethical and practical limitations given that the use of proprietary smartwatch data presents challenges in terms of accessibility and scalability. Widespread clinical adoption would require ensuring that similar physiological features can be derived from a broad range of devices, including more affordable or open-source alternatives. That said, our analytical framework is device-agnostic and could, in principle, be adapted to other wearables or consumer-grade biosensors. Our analysis included only participants with complete longitudinal data, which ensured data quality but may introduce adherence bias. Individuals who did not meet the minimum wearable or symptom-reporting requirements were excluded, potentially limiting generalizability to participants who are highly engaged or motivated to complete longitudinal monitoring.

To further evaluate the generalizability of our findings, validation efforts are ongoing in a prospective study to assess model performance in an independent cohort with longitudinal heart rate and symptom data. This prospective external validation will enable a stratified investigation across more diverse wearable devices and subgroups, including individuals with different vaccination statuses and demographic backgrounds, and would support an exploration of potential overlap with related syndromes such as myalgic encephalomyelitis/chronic fatigue syndrome (ME/CFS) or postural orthostatic tachycardia syndrome (POTS). However, due to the complex pathophysiology of Long COVID, elucidating the precise mechanisms underlying its effects on the cardiovascular system will remain challenging.

**Fig 4:**
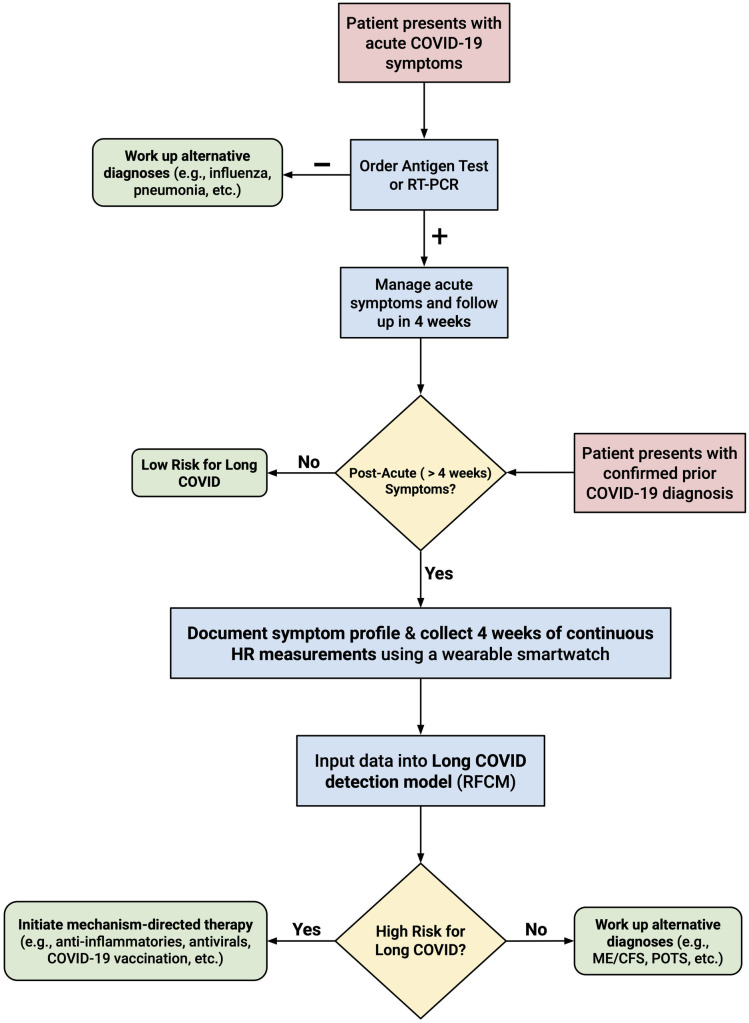
Clinical use algorithm. Flowchart displaying how a clinician might screen and manage a patient presenting with acute COVID-19 or a confirmed prior COVID-19 diagnosis using our Long COVID detection model.

This study demonstrates the efficacy of combining symptom data with longitudinal HR data — refined using step data — to diagnose Long COVID, persisting for up to 260 weeks, via machine learning. Our findings provide evidence that non-invasive, wearable-derived metrics can objectively complement clinical histories, potentially transforming existing diagnostic frameworks for Long COVID and meaningfully reducing misclassifications. Furthermore, the resulting Long COVID detection model we developed could be used by clinicians to guide clinical management (Fig 4). That is, the model could function as part of a clinical decision support system (CDSS), with the goal of flagging individuals at elevated risk of Long COVID and facilitating timely initiation of mechanism-directed therapy. While this study offers a proof of concept demonstrating the utility of wearable-derived metrics in Long COVID detection, future work will be needed to address challenges related to clinical integration, interpretability, and regulatory considerations to enable real-world applications. Ultimately, the granular decomposition of HR data into complex features may offer a pathway to better understanding the complex physiology underlying chronic conditions.

## Methods

### Study participants

In total, 5,571 adult individuals 18–80 years of age were recruited and consented for this study under protocol number 57022, approved by the Stanford University IRB. Participants were invited via social media, news coverage, and outreach to prior study cohorts. Participants registered using the REDCap survey system and were then asked to install the study app, called MyPHD (available for iOS and Android devices) [31]. The app transfers their wearable data (Fitbit via Fitbit secure OAuth 2.0 API; Apple Watch, Garmin, and other HealthKit-compatible devices via the HealthKit repository; and Google Fit-compatible devices) to a cloud platform for the Stanford research team to perform analysis. Participants were asked to wear their device for as long as possible both during the day and night. While participants wore the device, wearable data were transmitted to the MyPHD app on their smartphone via Bluetooth and did not require a continuous internet connection. Consent for the surveys was obtained through the secure REDCap survey system distributed via secure email, and consent for the use of wearable data and MyPHD was obtained through a secure REDCap link embedded in the MyPHD app. To protect participant privacy, each participant was assigned a random 22-digit ID number, and data were de-identified using double date-shifting by a unique random offset on the order of thousands of days.

Participants completed a comprehensive cross-sectional COVID-19 survey, which included questions characterizing the progression of their symptoms (S1 File). Additionally, they participated in longitudinal daily surveys, logging their symptom profiles throughout the course of their illness. Participants were labeled as Long COVID or non-Long COVID based on self-reported symptom duration, with Long COVID defined as the presence of symptoms 12 weeks or more (i.e., 3 months or more) after a confirmed COVID-19 diagnosis in accordance with the Centers for Disease Control and Prevention (CDC) [32]. Participants who reported experiencing Long COVID symptoms 12 weeks after their initial COVID-19 diagnosis date completed a final survey at the end of the study confirming the duration of their symptoms. Data were collected between March 10, 2020 and February 7, 2025.

From the dataset of 5,571 participants, 3,221 participants had usable data after cleaning and preprocessing, which included the exclusion of participants with incomplete survey data. Of these, 554 participants had a confirmed positive test for COVID-19 (e.g., a rapid antigen test or PCR test) and were included in the analysis for post-onset Long COVID detection, while the remaining 2,667 participants were COVID-negative.

The participant filtering process is illustrated in S7 Fig. Among the 554 COVID-positive participants, we further analyzed those with sufficient HR data. After applying additional filters to isolate resting HR metrics (i.e., removing activity-related noise using step data) and ensuring a consistent 4-week data period after a positive COVID test, 126 participants remained eligible for machine learning analysis.

### Heart rate processing

To mitigate the noise in the raw HR data due to physical activity, we leveraged step counts as an indicator of physical activity to filter out extraneous HR data. Specifically, we applied a 30-minute window following each recorded step, masking the HR data within these intervals. After this process, we aggregated the HR data by averaging over every 30-minute interval. This method ensured that we retained a comparable distribution of HR data during both sleep and wake periods, thus preserving the integrity of the dataset while effectively reducing noise.

We utilized the earliest viable four-week period of HR data to derive HR features. This period was defined as the earliest continuous interval of measurements requiring the fewest interpolations. If all four-week periods for a participant were missing greater than half of the data points, we excluded the participant from the analysis. To impute the missing 30-minute intervals, we used linear interpolation. This process resulted in the final 126 participants used for analysis. Features were derived across multiple categories, including time-domain analysis, Poincaré nonlinear analysis, raw signal analysis, Kullback-Leibler (KL) divergence, variational mode decomposition (VMD), and Shannon energy envelope (SEE). After all the features were derived from the 30-minute interval HR data over 4 weeks, we normalized each feature with a mean of 0 and a standard deviation of 1.

For time-domain analysis, we computed four features: the mean RR interval (MeanNN), the standard deviation of RR intervals (SDNN), the square root of the mean squared differences of successive RR intervals (RMSSD), and the proportion of successive RR interval differences exceeding 50 ms (pNN50). Poincaré nonlinear analysis included two features: SD1 (the standard deviation of successive RR intervals scaled by $\text{1}/\sqrt{\text{2}}$) and SD2 (the standard deviation of points along the line-of-identity axis). The RR intervals, representing the time between successive R-waves on an electrocardiogram (ECG), were estimated as the reciprocal of heart rate (60/HR).

Latent frequencies within the HR signal were extracted using Discrete Cosine Transforms (DCT). For a signal with *n* data points, the DCT decomposes it into a sum of *n* cosine functions, each with an associated coefficient. From the raw signal, we generated exactly 1,344 coefficients, with the first 45 coefficients accounting for a median of 85% of the signal’s variance. Intuitively, if a model responds well to a feature with DCT coefficient *k*, it means that the periodicity of the signal every *28/k* days is an important factor, since the total time period is 4 weeks or 28 days.

Daily RR interval distributions were reconstructed using kernel density estimation (KDE). KL divergence, which quantifies the difference between two probability distributions, was computed for each pair of daily distributions. From the resulting KL divergence matrix, we calculated the mean and standard deviation for each diagonal, representing day offsets from 1 to 28. These values were included as features summarizing temporal variations in RR interval distributions.

VMD decomposes a time series into modes, each representing a specific frequency band, by minimizing the bandwidth of each mode. For each of the five extracted modes (*u(i)*), we calculated statistical features such as mean, variance, skewness, kurtosis, primary frequency, and the correlation coefficient with the original signal. These features capture distinct physiological processes reflected in the HR signal.

The Shannon energy envelope (SEE) quantifies energy distribution across segments of the normalized HR signal modes derived from VMD. SEE has been shown to effectively identify changes in physiological signals, such as ECGs, while remaining robust to noise [33,34]. We computed the SEE for each VMD mode to evaluate energy dynamics across frequency bands. Following SEE computation, DCTs were applied again to convert the temporal sequence into discrete features suitable for the models.

### Data analysis

#### Symptom analysis.

To construct the symptom heatmap across the three phases of the COVID infection, we compiled survey data and stratified the results based on the relative duration since the confirmed COVID-19 diagnosis. For the acute phase (0–4 weeks after COVID-19 onset), we aggregated the counts of all symptoms reported by participants who completed surveys during this phase. Similarly, we computed aggregate symptom counts for the post-acute phase (4–12 weeks) and the chronic phase (12 weeks and beyond).

We calculated the expected symptom counts for each phase based on the total symptom counts across all phases and the total number of times each symptom was reported. Residual counts were calculated by subtracting these expected counts from the observed counts. By normalizing by the square root of the expected counts, we computed standard deviations from the expected values under the null hypothesis and constructed a matrix that displayed the divergence of symptom prevalence across COVID-19 phases.

### Model building and validation

We developed the Long COVID detection models using the random forest classifier from the scikit-learn package in Python. We selected the random forest algorithm due to its suitability for small datasets, robustness against overfitting, ability to handle heterogeneous feature types, and capacity to provide interpretable feature importance for our binary classification task. From a total of 313 HR and symptom features, we selected a subset of features through a two-step process. We set aside 40% of the data: 30% for feature selection and 10% for hyperparameter optimization. First, for heart rate features, we recursively eliminated features based on the random forest model’s feature importance calculation during training until half the features were eliminated. Feature importance was determined by the average reduction in impurity achieved across all trees in the forest. Because feature importance scores can be noisy with many features, we further evaluated the remaining features by performing 5-fold cross-validation on randomly selected groups of 6 features, repeating this grouping process for 40 trials. The performance of each feature across these trials was aggregated, and the 6 lowest-performing features were removed iteratively. To evaluate the impact of random chance on feature selection, we included a “random” feature drawn from a normal distribution. Features performing worse than this random feature were subsequently dropped, resulting in approximately 40 remaining features. From these, we identified the top seven symptom features and the top six HR features that maximized ROC-AUC performance after 5 randomized trials.

Random forest hyperparameters, including number of trees, maximum tree depth, and minimum samples per leaf, were optimized using randomized grid search on the 10% subset. Based on the final selected features and optimized model hyperparameters, we constructed three random forest models. The Random Forest Heart Rate Model (RFHRM) was trained using only HR features. The Random Forest Symptoms Model (RFSM) was trained using only symptom features. The Random Forest Combined Model (RFCM) was trained using both HR and symptom features.

The features used in the RFHRM include Raw Signal DCT 43, VMD Mode 4 SEE DCT 9, VMD Mode 0 SEE DCT 3, KL Divergence 15 Std, Raw Signal DCT 13, and Raw Signal DCT 36. The features used in the RFSM include acute chest pain, acute vomiting, post-acute excessive sweating, post-acute memory loss, post-acute brain fog, post-acute heart palpitations, and post-acute loss of smell. The features used in the RFCM include Raw Signal DCT 43, VMD Mode 4 SEE DCT 9, VMD Mode 0 SEE DCT 3, KL Divergence 15 Std, Raw Signal DCT 13, Raw Signal DCT 36, acute chest pain, post-acute memory loss, post-acute brain fog, post-acute heart palpitations, and post-acute loss of smell.

We evaluated the performance of each model by conducting 1,000 bootstrapped trials. For each trial, the dataset was re-randomized with replacement and split into training and test sets (70% and 30%, respectively). To account for class imbalance, train-test splits were stratified by Long COVID status to preserve class proportions in both sets. The 40% subset of the data that was used for feature selection was always included in the training set for each trial, and the 30% of the full dataset used for testing was randomly selected from the remaining 60% of the data. New classifiers were initialized for each trial, and the Receiver Operating Characteristic (ROC) and Precision-Recall (PR) curves were recorded. The mean Area Under the Curve (AUC) for the ROC and PR curves across the 1,000 trials was calculated, along with 95% confidence intervals. To compare the RFCM with the RFSM, we computed the paired differences in ROC-AUC and PR-AUC values for each trial and calculated the mean paired differences with 95% confidence intervals. To assess model overfitting, we extracted the mean ROC-AUC and mean PR-AUC values calculated using the train set and test set for comparison.

### Case study

We conducted a case study to compare the performance of the Random Forest Combined Model (RFCM) and the Random Forest Heart Rate Model (RFHRM) by training versions of both models that represented the average performance of the bootstrapped trials with the same training set (n = 88) and evaluating them on a shared test set (n = 38) after setting classification thresholds that optimized the F1 score for each trained model. We calculated the sensitivity and specificity of each of these models for classifying Long COVID in the test set. To assess model interpretability, we applied SHapley Additive exPlanations (SHAP) using the shap package in Python.

### Statistical analysis

Statistically significant standard deviations in the symptom prevalence heatmap (Fig 2) were determined by transforming the computed deviation for each symptom and phase into p-values using the standard normal cumulative distribution function (CDF) (μ = 0, σ = 1). Statistical significance was defined as *P* < 0.05 after adjusting for multiple comparisons using the Benjamini-Hochberg procedure with a false discovery rate (FDR) at 5%.

We summarized the demographic and baseline characteristics of our cohort, stratifying by those with and without Long COVID. Independent t-tests were performed to compare each characteristic between the two subgroups, with statistical significance being defined as *P* < 0.05 after adjusting for multiple comparisons using the Benjamini-Hochberg procedure with a false discovery rate (FDR) at 5%.

All analyses were conducted using Python software version 3.12.3.

## Supporting information

S1 Fig Time-aligned trajectories of resting heart rate and symptoms in representative participants. Plots illustrating resting heart rate (RHR) trajectories aligned to the date of COVID-19 diagnosis (Week 0, red line) with reported symptoms annotated for acute and post-acute phases. **(a)** Representative participant with Long COVID **(b)** Representative participant without Long COVID.
(TIFF)

S2 Fig Distribution of participant 4-week data period start times. Histogram (n = 126) of the start time of the earliest viable four-week data period for all participants. The earliest viable four-week period was determined as the earliest period requiring the fewest data interpolations.
(TIFF)

S3 Fig Distribution of duration of Long COVID. Box plot (*n = *31) showing the median duration of Long COVID in weeks (horizontal line), interquartile range (box), and data range within 1.5 times the interquartile range (whiskers); individual data points are shown as dots, illustrating the variability and density of durations across the Long COVID subset.
(TIFF)

S4 Fig Train vs. test performance metrics. **(a)** Bar chart showing the average ROC-AUC from the train vs test sets for the Random Forest Combined Model (RFCM), Random Forest Symptoms Model (RFSM), and Random Forest Heart Rate Model (RFHRM). **(b)** Bar chart showing the average PR-AUC from the train vs test sets for the RFCM, RFSM, and RFHRM. Error bars indicate the standard deviation of the average ROC-AUC or PR-AUC.
(TIFF)

S5 Fig Case study confusion matrices. **(a)** Confusion matrix for the Random Forest Symptoms Model (RFSM), showing the model’s predictions for the case study test set based on symptom-derived features alone. **(b)** Confusion matrix for the Random Forest Combined Model (RFCM), which integrates both symptom-derived features and heart rate (HR) features.
(TIFF)

S6 Fig SHAP Summary Plot. Beeswarm plot showing feature impact and analysis for the case study Long COVID participants (n = 3) that were misclassified by the Random Forest Symptoms Model (RFSM) but correctly classified by the Random Forest Combined Model (RFCM).
(TIFF)

S7 Fig Study participant selection. A flow chart over the participants in the study, including the number of COVID-19 positive cases and COVID-19 negative cases as well as the number of COVID-19 positive cases with sufficient heart rate data for analysis. The COVID-19 positive cases are further stratified into those who reported Long COVID and those who did not.
(TIFF)

S1 File Long COVID questionnaire. The cross-sectional COVID-19 survey, including questions characterizing the progression of their symptoms, administered to study participants.
(PDF)

S2 File Wearable data time windows. A table displaying four-week time windows, from Start Week to End Week after an initial COVID-19 diagnosis, used to preprocess each participant’s wearable data before machine learning analysis.
(XLSX)

S3 FileDerived heart rate features.A table displaying the 202 unique features derived from each participant’s heart rate data across six analytics categories. SEE, Shannon Energy Envelope; DCT, Discrete Cosine Transform; NN, RR interval; SDNN, Standard Deviation of the RR interval; RMSSD, square root of the mean squared differences of successive RR intervals; pNN50, the proportion of successive RR interval differences exceeding 50 ms; SD1, the standard deviation of successive RR intervals scaled by $\text{1}/\sqrt{\text{2}}$; SD2, the standard deviation of points along the line-of-identity axis; kl div, Kullback-Leibler divergence.(XLSX)

S4 File Bootstrapped trial results. A table displaying the ROC-AUC and PR-AUC values corresponding to the Random Forest Combined Model (RFCM), Random Forest Symptoms Model (RFSM), and Random Forest Heart Rate Model (RFHRM) for each of the 1000 bootstrapped trials.
(XLSX)
