# Supplementary material for: Longitudinal wearable sensor data enhance precision of Long COVID detection
Source: PLOS Digit Health. 2025 Nov 20;4(11):e0001093. doi: 10.1371/journal.pdig.0001093 (PMC12633932; doi:10.1371/journal.pdig.0001093)
Supplement: S1 File — The cross-sectional COVID-19 survey, including questions characterizing the progression of their symptoms, administered to study participants. (PDF) [file pdig.0001093.s008.pdf]

COVID-19 Infection Survey

Please use this survey to describe a single COVID-19 infection. If you have had multiple COVID-19 infections, please fill out a separate instance of this survey for each infection.

After you submit this survey, you can later edit your responses to report new information for this infection.

Was your infection confirmed by a positive SARS-CoV-2 PCR or Rapid Antigen Test (Home based kit) ?

- ◯ Yes
- ◯ No
- ◯ I don't know

What was the date of your first positive test confirming this infection?

____

If you have an estimate for the most likely date of exposure leading to this infection, please enter it here.

____

Select all locations where you were treated or sought treatment for COVID-19.

- ☐ Emergency room or urgent care
- ☐ Outpatient clinic
- ☐ Inpatient ward
- ☐ Inpatient intensive care unit (ICU)
- ☐ Home based care
- ☐ None of the above

Please enter the date of your emergency room or urgent care visit. If you had multiple urgent care visits, please enter the first date.

____

Please enter the date of your outpatient clinic visit. If you had multiple outpatient clinic visits, please enter the first date.

____

What was your date of hospital admission?

____

What was the date of ICU admission ?

____

When did you shift out from the ICU to a regular ward?

____

Have you been discharged from the hospital?

- ◯ Yes
- ◯ No

What was your date of hospital discharge?

____

What was the severity of your COVID-19 infection?

- ◯ Asymptomatic
- ◯ Mild (experienced symptoms, but no shortness of breath or abnormal chest radiological imaging)
- ◯ Moderate (pneumonia (infection of lungs) and/or abnormal chest radiological imaging)
- ◯ Severe (pneumonia with blood oxygen saturation levels less than 94 % or breathing rate > 30 breaths/min or requiring care in the ICU)
- ◯ Critical (required some form of assisted ventilation in the ICU)

Have you received any of the following diagnoses by a healthcare professional?	☐ Post Acute COVID-19<br>☐ Chronic Post COVID-19 Syndrome<br>☐ "Long COVID"<br>☐ "Chronic COVID"<br>☐ Post-COVID syndrome (PCS)<br>☐ Post-acute sequelae of SARS-CoV-2 infection (PASC)<br>☐ Persistent symptoms after acute SARS-CoV-2 infection<br>☐ None of the above
What was the date of this diagnosis?	______________________

We distinguish multiple phases of COVID-19, which we have defined as the following:

Acute COVID-19
Symptoms within 4 weeks of COVID-19 onset.

Post Acute COVID-19
Symptoms from 4 to 12 weeks after COVID-19 onset that are not explained by any other diagnoses.

Chronic Post COVID-19 Syndrome
Symptoms 12 weeks after COVID-19 onset that are not explained by any other diagnoses.

Have you experienced any Acute COVID-19 symptoms?	◯ Yes<br>◯ No
When did your Acute COVID-19 symptoms start?	______________________<br>(If you are unsure, enter your best estimate.)
Are you currently experiencing Acute COVID-19 Symptoms?	◯ Yes<br>◯ No
When did your Acute COVID-19 symptoms end?	______________________<br>(If you are unsure, enter your best estimate.)
Have you experienced any Post Acute COVID-19 symptoms?	◯ Yes<br>◯ No
When did your Post Acute COVID-19 symptoms start?	______________________<br>(If you are unsure, enter your best estimate.)
Are you currently experiencing Post Acute COVID-19 Symptoms?	◯ Yes<br>◯ No
When did your Post Acute COVID-19 symptoms end?	______________________<br>(If you are unsure, enter your best estimate.)
Have you experienced any Chronic Post COVID-19 Syndrome symptoms?	◯ Yes<br>◯ No
When did your Chronic Post COVID-19 Syndrome symptoms start?	______________________<br>(If you are unsure, enter your best estimate.)

Are you currently experiencing Chronic Post COVID-19 Syndrome Symptoms?
◯ Yes
◯ No

When did your Chronic Post COVID-19 Syndrome symptoms end?
____
(If you are unsure, enter your best estimate.)

From the systematic list provided below, please select all symptoms that you experienced during each of the aforementioned infection periods

Systemic symptoms

	Acute COVID-19 [within 4 weeks of COVID-19 detection]	Post Acute COVID-19 [from 4 to 12 weeks after COVID-19 detection]	Chronic Post COVID-19 Syndrome [12 weeks after COVID-19 detection]
Fever (>99 ºF)	☐	☐	☐
Fatigue	☐	☐	☐
Weight loss	☐	☐	☐
Decreased tolerance for exercise/exertion	☐	☐	☐
Chills or night sweats	☐	☐	☐
Muscle / joint / body aches	☐	☐	☐

Respiratory

	Acute COVID-19 [within 4 weeks of COVID-19 detection]	Post Acute COVID-19 [from 4 to 12 weeks after COVID-19 detection]	Chronic Post COVID-19 Syndrome [12 weeks after COVID-19 detection]
Dry cough	☐	☐	☐
Cough with mucus production	☐	☐	☐
Sneezing / runny nose	☐	☐	☐
Sore throat	☐	☐	☐
Shortness of breath / difficulty breathing	☐	☐	☐

Neuropsychiatric

	Acute COVID-19 [within 4 weeks of COVID-19 detection]	Post Acute COVID-19 [from 4 to 12 weeks after COVID-19 detection]	Chronic Post COVID-19 Syndrome [12 weeks after COVID-19 detection]
Headache	☐	☐	☐
Cognitive dysfunction / slowed thinking / brain fog	☐	☐	☐
Sudden altered mental status / disorientation / confusion	☐	☐	☐
Memory issues	☐	☐	☐
Sleep disturbances / insomnia	☐	☐	☐

Anxiety / depression	☐	☐	☐

Special senses

	Acute COVID-19 [within 4 weeks of COVID-19 detection]	Post Acute COVID-19 [from 4 to 12 weeks after COVID-19 detection]	Chronic Post COVID-19 Syndrome [12 weeks after COVID-19 detection]
Loss of smell / altered smell	☐	☐	☐
Loss of taste / altered taste	☐	☐	☐
Hearing issues / ringing sensation	☐	☐	☐
Vision disturbances / blurring	☐	☐	☐
Abnormal sensations / pins and needles	☐	☐	☐

Gastrointestinal

	Acute COVID-19 [within 4 weeks of COVID-19 detection]	Post Acute COVID-19 [from 4 to 12 weeks after COVID-19 detection]	Chronic Post COVID-19 Syndrome [12 weeks after COVID-19 detection]
Nausea / vomiting	☐	☐	☐
Abdominal pain	☐	☐	☐
Acid reflux	☐	☐	☐
Diarrhea	☐	☐	☐
Constipation	☐	☐	☐
Loss of appetite	☐	☐	☐
Irritable bowel syndrome	☐	☐	☐

Cardiovascular

	Acute COVID-19 [within 4 weeks of COVID-19 detection]	Post Acute COVID-19 [from 4 to 12 weeks after COVID-19 detection]	Chronic Post COVID-19 Syndrome [12 weeks after COVID-19 detection]
Palpitations / faster, pounding, or irregular heart beat	☐	☐	☐
Dizziness on standing from lying down or sitting positions	☐	☐	☐
Chest pain (sharp/aching/burning)	☐	☐	☐
Lightheadedness / fainting episodes	☐	☐	☐

Dermatologic

	Acute COVID-19 [within 4 weeks of COVID-19 detection]	Post Acute COVID-19 [from 4 to 12 weeks after COVID-19 detection]	Chronic Post COVID-19 Syndrome [12 weeks after COVID-19 detection]
Pale, gray or bluish discoloration of skin, lips, nail beds, fingers or toes	☐	☐	☐

Rashes / itchiness	☐	☐	☐
COVID toe / swelling, discoloration, or rash on toe	☐	☐	☐

Reproductive

	Acute COVID-19 [within 4 weeks of COVID-19 detection]	Post Acute COVID-19 [from 4 to 12 weeks after COVID-19 detection]	Chronic Post COVID-19 Syndrome [12 weeks after COVID-19 detection]
Menstrual irregularities / period problems	☐	☐	☐
Sexual dysfunction	☐	☐	☐

Flare up of pre-existing conditions

	Acute COVID-19 [within 4 weeks of COVID-19 detection]	Post Acute COVID-19 [from 4 to 12 weeks after COVID-19 detection]	Chronic Post COVID-19 Syndrome [12 weeks after COVID-19 detection]
Chronic Obstructive Pulmonary Disease (COPD)	☐	☐	☐
Asthma	☐	☐	☐
Allergies	☐	☐	☐
Diabetes	☐	☐	☐
Thyroid dysfunction	☐	☐	☐
Hypertension	☐	☐	☐
Increased blood cholesterol	☐	☐	☐
Kidney disease	☐	☐	☐
Liver disease	☐	☐	☐
Rheumatoid arthritis	☐	☐	☐
Autoimmune disorders	☐	☐	☐

If you experienced any other Acute COVID-19 symptoms, please list them here. ______________________________

If you experienced any other Post Acute COVID-19 symptoms, please list them here. ______________________________

If you experienced any other Chronic Post COVID-19 symptoms, please list them here. ______________________________

Describe the symptom severity during each phase of infection.

	Acute COVID-19 [within 4 weeks of COVID-19 detection]	Post Acute COVID-19 [from 4 to 12 weeks after COVID-19 detection]	Chronic Post COVID-19 Syndrome [12 weeks after COVID-19 detection]
Symptoms did not prevent me from performing any everyday activities	☐	☐	☐
Occasionally needed to avoid or reduce everyday activities	☐	☐	☐
Unable to perform everyday activities	☐	☐	☐
Dependent on nursing care or assistance from others	☐	☐	☐
Sought care from a healthcare professional	☐	☐	☐
Warranted hospital or emergency room admission	☐	☐	☐

Please enter all that apply:

Post Acute COVID-19 Chronic Post COVID-19 Syndrome
Hospital Admission Date ______ ______
Hospital Discharge Date ______ ______
Reason for hospital/emergency room admission ______ ______

Questions about personal history

Do you currently smoke ?
◯ Yes
◯ No

Have you ever smoked ?
◯ Yes
◯ No

Which year did you start smoking ?
____

Which year did you stop smoking ?
____

On average how many cigarettes per day do/did you smoke ?
____

Vaccination questions

Please enter company and dates for all of your vaccine doses (including boosters).

Please enter all vaccine doses or boosters you have had:

Dose/Booster 1 ______ ______
Dose/Booster 2 ______ ______
Dose/Booster 3 ______ ______
Dose/Booster 4 ______ ______
Dose/Booster 5 ______ ______
Dose/Booster 6 ______ ______
